# Large regional variability in geomagnetic storm effects in the auroral zone

**DOI:** 10.1038/s41598-023-46352-0

**Published:** 2023-11-02

**Authors:** Otto Kärhä, Eija I. Tanskanen, Heikki Vanhamäki

**Affiliations:** 1 Sodankylä Geophysical Observatory, University of Oulu, Tähteläntie, 99600 Sodankylä, Finland; 2 Department of Electronics and Nanoengineering, Aalto University, Maarintie, 02150 Espoo, Finland; 3 Space Physics and Astronomy Research Unit, University of Oulu, Pentti Kaiteran Katu, 90570 Oulu, Finland

**Keywords:** Aurora, Magnetospheric physics

## Abstract

A digital society is fragile and vulnerable to space-originated electromagnetic disturbances. Global geomagnetic conditions have been actively monitored since the invention of the magnetometer in 1833. However, regional changes in the magnetic environment have been widely left unstudied because of the sparsity of the observing networks. The Scandinavian Magnetometer Array (SMA) was the densest magnetometer network in history, and it was in operation in Fennoscandia during the International Magnetospheric Study (IMS) in 1976–1979. The data has been left mainly unstudied because it was recorded on 35 mm films, which are difficult to use for scientific studies. We used the DigiMAG digitization method to digitize magnetic data from all 32 SMA stations for a geomagnetic storm on 10–12 December 1977. Using these digitized values and modern magnetic data, we found large regional differences about up to 2 nT/km during strong geomagnetic storms (Dst 100–200 nT) and 7 nT/km for major scale Halloween geomagnetic storm, which correspond to 400 and 1400 nT difference for a typical 200 km station separation, respectively. The average size of substorms is 400 nT in the auroral zone. We conclude that the sparse magnetometer network can cause an underestimation of the regional magnetic disturbances and their effects. Misestimation of regional disturbances during extreme storms like the Carrington event may lead to insufficient planning of mitigation procedures and strategies.

## Introduction

Geomagnetic conditions have been measured in the Scandinavian region for a long time with compasses, dip meters and magnetometers. An interrelation between aurorae and geomagnetic disturbances was discovered in the 1740s^1,2^. Still today, understanding of the spatial variability of magnetic disturbances in the auroral zone is largely lacking due to the limitations of the observing infrastructures. We study this spatial variability in the north component of the magnetic variation field in this region using the newly digitized data from the Scandinavian Magnetometer Array (SMA) network and modern geomagnetic data from the Sodankylä Geophysical Observatory (SGO) and International Monitor for Auroral Geomagnetic Effects (IMAGE) network^3^. The data used includes two strong geomagnetic storms with the lowest Disturbance Storm Time (Dst) index intensities of − 159 nT (28 October 1977) and − 112 nT (10–12 December 1977) and one great geomagnetic storm with a peak intensity of − 353 nT (28–31 October 2003). This study aims to better understand regional geomagnetic activity differences in the auroral zone during the studied geomagnetic storms and to find the largest spatial differences in the north component of the variation field as nT/km values. We have selected magnetometer station pairs for both magnetometer networks to present the lower limits for the regional differences in the magnetic environment at the Fennoscandian auroral region with the measured data.

Geomagnetic storms are identified as strong if their Dst peak intensity is between − 100 and – 200 nT, severe storms when this peak intensity is between − 200 and − 350 nT, and great storms when this maximum intensity decreases below − 350 nT^4^. For this paper, we have digitized data on 10–12 December 1977 (Dec77 from this onwards) from all 32 SMA magnetometers, which we compare to the earlier digitized data for the most intense geomagnetic storm of the year 1977, namely 28 October 1977 (Oct77)^5^, and Halloween storm on 28–31 October 2003 (Oct03). The year 1977 was at the early ascending phase of the solar cycle 21 which had pronounced peaks in June and October, and it is reported to be a solar northern hemisphere-dominated year^6^, while the year 2003 was part of the early declining solar cycle phase and it was the most active year in the last century^7^.

Geomagnetic disturbances in the auroral zone can grow to the size of thousands of nanoteslas during geomagnetic storms^3^. The largest substorm intensity we found in this study is − 4434 nT (see the last paragraph before Discussion). Large geomagnetic storms cause variations in the thermospheric and ionospheric plasma density^8,9^ and induce currents to the ground^10^, which can cause malfunctions in high-tech systems. Ground induced currents can be studied by separating the externally and internally driven parts^11–13^, and thus showing how large portion of the solar-originated energy is used to drive ground induced currents. Typically, 10–25% of the total dissipated energy is dissipating in a form of ground induced currents^11^.

## Auroral zone variability during the strong geomagnetic storm

**Figure 1:**
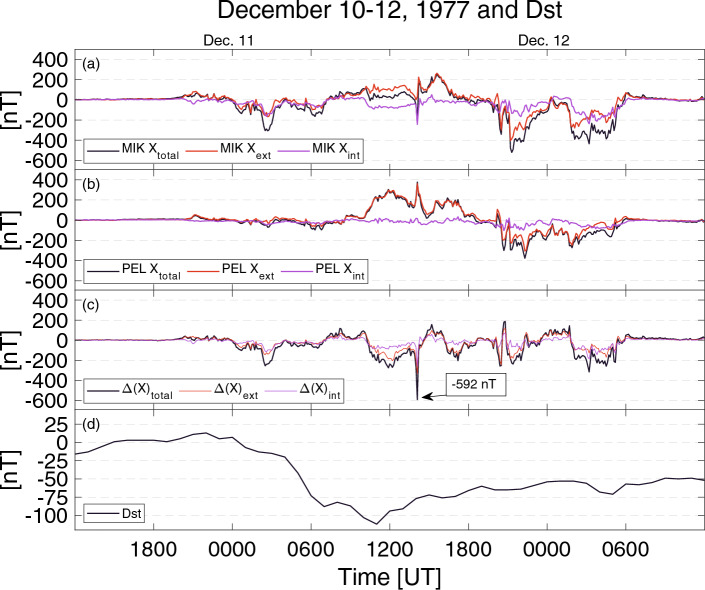
Digitized north–south component of the magnetic field and Dst. (**a**) Mikkelvik (MIK) and (**b**) Pello (PEL). The difference of MIK and PEL total, external, and internal variation field (**c**). The Disturbance Storm Time (Dst) index (**d**).

Newly digitized data for the 10–12 December 1977 geomagnetic storm show westward and eastward auroral electrojets, strong geomagnetic disturbances up to 636 nT in magnitude and a large momentary difference between the stations. The event was the third-largest geomagnetic storm of that year, and data from 32 SMA stations was available and digitized for this event. The Dst peak amplitude reached − 112 nT (Fig. 1d), and thus, it is categorized as a strong geomagnetic storm^4^.

The largest difference measured by the SMA network was seen on the Dec77 storm at 1406 UT between Rostadalen (ROS, Lat. 68.97° Long. 19.67°) and Mikkelvik (MIK, Lat. 70.07° Long. 19.03°) station pair. The largest difference was 2.4 nT/km (in total 296 nT), which was observed for the station separation of 125 km. The typical variability in the magnetic environment in the auroral zone was seen between Mikkelvik and Pello (PEL, Lat. 66.85° Long. 24.73°) stations, which are 426 km apart. Figure 1a,b presents the digitized north–south component (X-component) from Mikkelvik and Pello station recordings. Mikkelvik recorded a small westward electrojet of − 215 nT and Pello station eastward electrojet with an amplitude of 377 nT. These values give a momentary difference of 592 nT (1.4 nT/km) in the north–south magnetic field component at 1406 UT (Fig. 1c). Note that the largest instantaneous differences between MIK and PEL are seen in the afternoon sector instead of late evening or early morning sectors, where the typical substorm signatures are seen.

In our previous study, regional differences in magnetic activity at the northern Fennoscandia for the Oct77 geomagnetic storm were found to be able to exceed 500 nT over a distance of 167 km^5^. This result gives regional differences of around 3 nT/km between the observing stations for the strong geomagnetic storm with the Dst index 159 nT. The magnetic field can be separated into external and internal parts^12,13^. The external part is shown with a red curve in Fig. 1a–c and the induced part with a purple color. The internal part describes how strong currents are induced to the ground during the observed magnetic disturbances. The induced part is about 10–50% for the coastal station MIK, while it is much smaller, around 5–20% for the inland station PEL.

This result indicates that only a portion of the energy carried by the geomagnetic disturbances enters to induced currents and that there are large regional differences in how geomagnetic storms affect the vulnerable infrastructures such as pipelines and electrical grids. The finding agrees with the previous results^11^, where the largest induced currents were reported to be seen close to the seas and during rapid changes in the geomagnetic environment such as substorm onsets.

**Figure 2:**
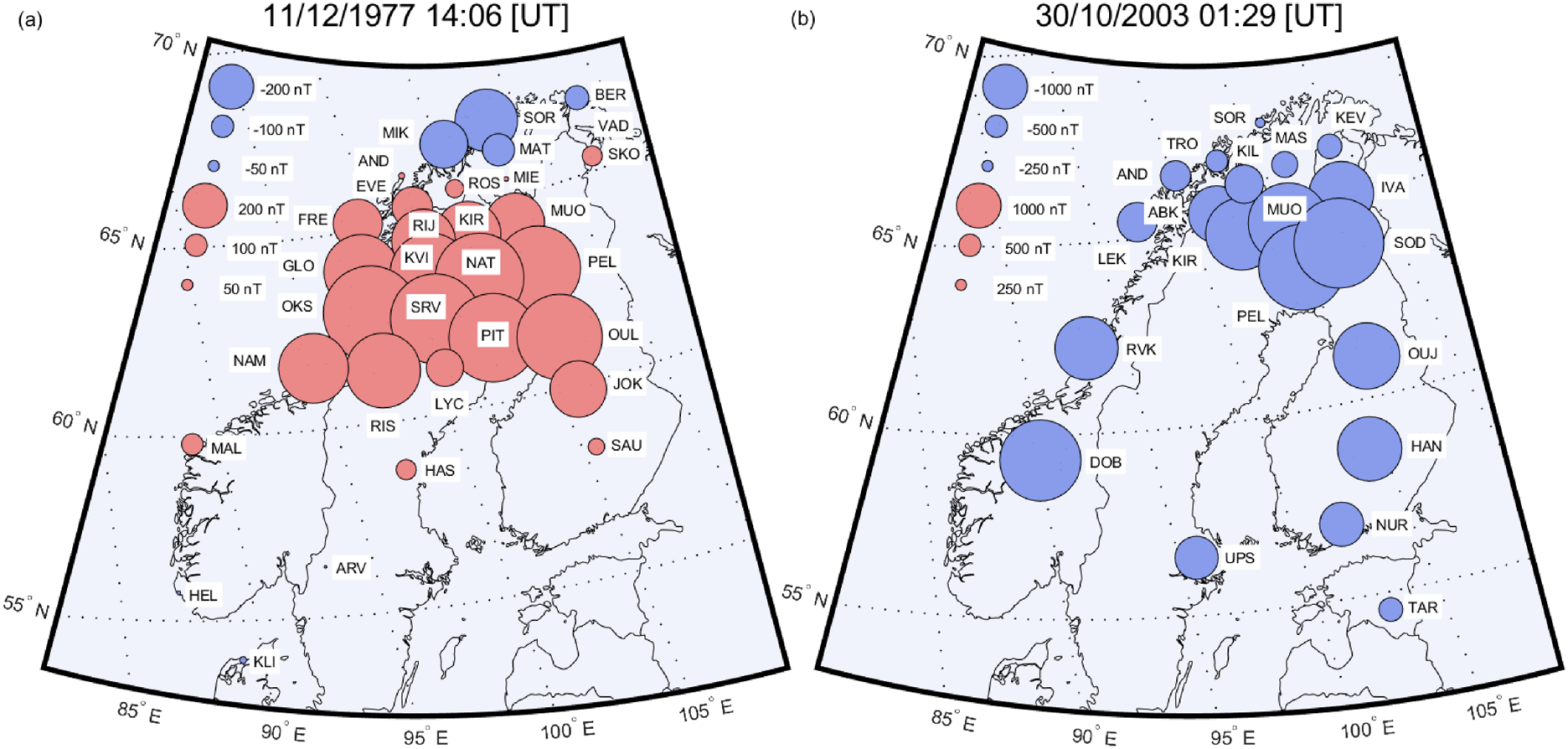
The magnetic environment in Scandinavia during major nT/km differences. (**a**) Digitized SMA X-component data disturbances for time 1406 UT (**b**) IMAGE network X-component disturbances 30/10/2003 01:28:30 UT. The CGM coordinates for the studied storms are marked in the images.

Figure 2a shows the digitized data for all SMA stations for the Dec77 event at 1406 UT when the largest difference between MIK and PEL stations in the studied northern component of the variation field occurs. The strength of the eastward electrojet is shown with the red circles, and the strength of the westward electrojet with the blue circles. The disturbances between 60° and 65° (corrected geomagnetic coordinates (CGM)^14^) are 167 to 423 nT stronger than before the storm period 10 December at 1200 UT. The borderline between eastward and westward electrojets is seen around 67°, which is clearly visible because of the SMA network’s dense and regular station spacing. This latitude matches with the previous studies for 2014–2017 with the Kp index larger than 2, which have shown that the divergence-free currents are distributed on average around 70° AACGM (altitude-adjusted corrected geomagnetic coordinates)^15^. Note that the CGM latitude has shifted about one degree north since 1977^14,16^.

## Auroral zone variability during the great geomagnetic storm

An exceptionally powerful series of geomagnetic storms, called Halloween storms, occurred between late October and early November 2003. Continuous fast solar wind erupted from coronal holes, and over 80 coronal mass ejections were detected during a three-week period^17^. The fastest speed observed by Advanced Composition Explorer (ACE spacecraft) reached 1850 km/s on 29 October^18^, which is more than three orders of magnitude faster than a bullet. The same day, the Dst index decreased to − 353 nT, and the storm is classified as a great geomagnetic storm based on this peak intensity^4^.

Figure 2b shows the geomagnetic disturbances during the Oct03 storm at 01:29 UT when the largest spatial difference per kilometer between the IMAGE stations was 7.6 nT/km (in total 1210 nT), detected between Masi (MAS, Lat. 69.46° Long. 23.70°) and Muonio (MUO, Lat. 68.02° Long. 23.53°) stations separated by 160 km. The average maximum difference between all IMAGE station pair combinations (180 pcs.) was 1684 nT or 3.34 nT/km. Thus, Sodankylä (SOD, Lat. 67.37° Long. 26.63°) – Tromso (TRO, Lat. 69.66° Long. 18.94°) station pair represents typical differences at the auroral region with maximum difference 1514 nT, which gives 3.8 nT/km with the 403 km station separation.

Exceptionally large magnetic disturbances were detected during the Halloween storm throughout the observing network in Fennoscandia. The largest disturbances due to the westward electrojets (i.e. negative bays) were detected in Oulujärvi, where − 4434 nT was recorded, and − 1982 nT disturbances were seen as south as Tartu. The largest magnetic disturbances due to the eastward electrojet (i.e. positive bays) varied between Sørøya (1031 nT) and Leknes (634 nT). These findings are consistent with previous research^3^ and underline the importance of the knowledge of regional geomagnetic disturbances.

## Discussion

The Carrington Event occurred in 1859 and is still the strongest recorded storm^19^. It has been described that the surface of the Sun momentarily brightened significantly and dazzled the protected eye^20^. The report mentions that the magnetic storm induced currents in telegraph lines, which led to arcing, igniting fires^21^. It is estimated that during this particular storm, the Dst index decreased to − 1760 nT^22^. Recent studies have found similarities between the Carrington event and the Oct03 storm^23,24^. The rarity of great geomagnetic storms limits the study of the extreme geomagnetic events and their regional effect. Thus, the data recorded during the Oct03 storm with modern magnetometers is so valuable. We studied the regional spatial variability of the north component of the magnetic variation field in the auroral zone using digitized values from the SMA network together with the other available magnetic data.

**Figure 3:**
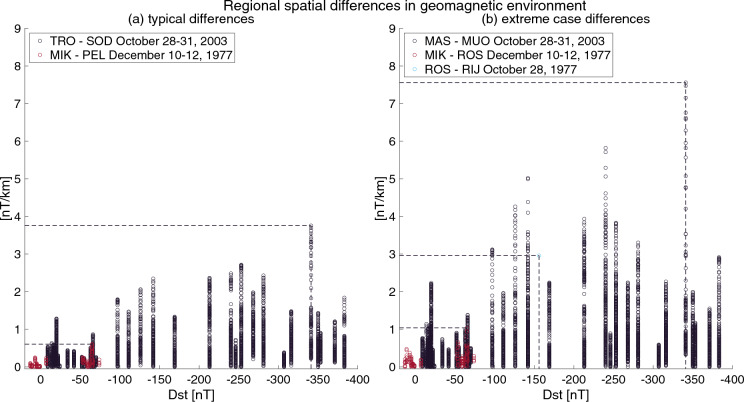
Regional spatial differences in geomagnetic environment. (**a**) Typical differences on the night side, and (**b**) extreme case differences, respectively.

The results show greater station-to-station differences in north–south direction than previously reported. The largest difference (1210 nT or 7.6 nT/km) appeared between Masi (MAS, Lat. 69.46° Long. 23.70°) and Muonio (MUO, Lat. 68.02° Long. 23.53°) during the Oct03 storm. Geomagnetic activity in the auroral zone and geomagnetic storms at the equator are closely related. Formerly, it was believed that magnetic storms with an associated ring current could be a superposition of successive substorms. In this model, the ring current of a magnetic storm would be constructed by successive particle injections associated with intense substorms^25,26^. Thus, comparing local variations and finding connections with the Dst index measured relatively close to the equator is appropriate. Figure 3 shows the differences between selected station pairs in the nT/km scale. Regional differences in geomagnetic activity during Oct77, Dec77 and Oct03 geomagnetic storms are marked with red, blue, and black circles. The extreme peak value for regional differences on the night side for Dec77 storm is around 1.0 nT/km, for Oct77 around 3.0 nT/km, for Oct03 around 7.6 nT/km, and they increase nearly linearly according to the storm intensity (Dst-index − 112, − 159 and − 353 nT respectively). The typical values are slightly lower for Oct03 and Dec77 storms (0.61 and 3.80, respectively).

When we consider these rapidly increasing peak values, the digitized and other magnetic data presented in this paper can be used to give an order of magnitude estimates on the regional magnetic disturbances in the aurora zone during extreme solar storms such as Carrington and Miyake^27,28^ events. When linear extrapolation is used for estimating the maximum amplitude, regional disturbances can possibly grow up to 15 nT/km for the Carrington-scale events. The similar extrapolation would give even two times larger regional gradients for the Miyake-scale events. For such extreme event amplitude estimation, it needs to be remembered that there is expected to be an upper limit, or saturation, for how large the magnetic disturbances can grow^29^. The saturation for the regional magnetic disturbances in the auroral zone can be further studied with the new methods and digitized data.

## Methods

### SMA data

The Scandinavian Magnetometer Array recorded magnetic field disturbances with (HDZ-component) representation, and the X-component can be calculated as X = H cos(Ddeg) – Dnt sin(Ddeg). The instruments were based on modified Gough-Reitzel magnetometers with three wire-suspended magnets and a camera. These Type Münster magnetometers were suitable for the measurements in the frequency range from 10 μHz to 20 mHz^30–32^. The total number of SMA stations was 36, of which four were installed after 1977. 32 SMA stations recorded the studied December 1977 storm. Sodankylä Geophysical Observatory has the original recordings, and the dataset contains more than 600 magnetograms. A custom-built digitization device has been developed primarily to photograph these 35 mm tapes. Data must be collected from photographs manually and scaled according to the values of the magnetogram. Our previous study describes the digitization method in more detail^5^. We want to encourage researchers to digitize and publish historical data before it becomes fragile and unreadable.

## Data Availability

Photographs of the studied SMA magnetograms and digitized numerical values are available at https://doi.org/10.23729/99d49197-cb06-4e2e-aa7f-ce5311eaa2ed.
